# The Boltysh impact structure: An early Danian impact event during recovery from the K-Pg mass extinction

**DOI:** 10.1126/sciadv.abe6530

**Published:** 2021-06-18

**Authors:** Annemarie E. Pickersgill, Darren F. Mark, Martin R. Lee, Simon P. Kelley, David W. Jolley

**Affiliations:** 1 School of Geographical and Earth Sciences, University of Glasgow, Gregory Building, Lilybank Gardens, Glasgow, UK.; 2 Scottish Universities Environmental Research Centre, East Kilbride, UK.; 3 Department of Earth and Environmental Science, University of St Andrews, St Andrews, UK.; 4 School of Earth and Environment, Faculty of Environment, University of Leeds, Leeds, UK.; 5 Geology and Petroleum Geology, School of Geosciences, University of Aberdeen, Aberdeen, UK.

## Abstract

Both the Chicxulub and Boltysh impact events are associated with the K-Pg boundary. While Chicxulub is firmly linked to the end-Cretaceous mass extinction, the temporal relationship of the ~24-km-diameter Boltysh impact to these events is uncertain, although it is thought to have occurred 2 to 5 ka before the mass extinction. Here, we conduct the first direct geochronological comparison of Boltysh to the K-Pg boundary. Our ^40^Ar/^39^Ar age of 65.39 ± 0.14/0.16 Ma shows that the impact occurred ~0.65 Ma after the mass extinction. At that time, the climate was recovering from the effects of the Chicxulub impact and Deccan trap flood volcanism. This age shows that Boltysh has a close temporal association with the Lower C29n hyperthermal recorded by global sediment archives and in the Boltysh crater lake sediments. The temporal coincidence raises the possibility that even a small impact event could disrupt recovery of the Earth system from catastrophic events.

## Teaser

The Boltysh impact structure formed 0.65 Ma after the K-Pg boundary, coincident with the Lower C29n hyperthermal.

## INTRODUCTION

Perturbations to the Earth system leading to rapid environmental change can lead to mass extinctions and longer-term ecological and biotic effects. Postextinction recovery of ecosystems is characterized by complex dynamics operating over periods of 100s ka to >1 Ma (*1*). The Phanerozoic has seen five major mass extinctions [e.g., (*2*)], most recently at the Cretaceous-Paleogene (K-Pg) boundary, 66.04 ± 0.02/0.08 Ma [2σ, analytical/systematic uncertainty, as determined by the ^40^Ar/^39^Ar age of the stratigraphically nearest datable sanidine, the Iridium-Z (IrZ) sanidine (*3*, *4*)]. It has been estimated that the K-Pg mass extinction resulted in a 50% loss of terrestrial species and a 75% loss of marine species (*5*–*7*) and served as a biological filter that profoundly affected the nature and structure of modern ecosystems (*8*).

The K-Pg mass extinction is linked to a hypervelocity impact at 66.04 ± 0.05/0.10 Ma [2σ, analytical/systematic uncertainty, as determined by ^40^Ar/^39^Ar geochronology of glass spherules (*4*, *9*)] that produced the ~200-km-diameter Chicxulub impact structure in Mexico. This event occurred during the eruption of the Deccan Traps Large Igneous Province (*10*), which is proposed to have contributed to latest Cretaceous and possibly earliest Paleocene environmental change and biological turnover (*10*–*12*). The end phase of Deccan Trap volcanism continued until at least 65.27 ± 0.12/0.15 Ma [2σ, analytical/systematic (*13*)].

In addition to Chicxulub, a lesser-known impact structure is temporally associated with the K-Pg boundary—the Boltysh impact structure, Ukraine (*14*). Previous ^40^Ar/^39^Ar geochronology yielded an age for Boltysh of 65.80 ± 0.64/0.68 Ma [2σ, analytical/systematic uncertainty (*14*), recalculated using the optimization model of (*15*) and the parameters of (*16*)]. This age is insufficiently precise to temporally distinguish the two impacts from each other or from the K-Pg boundary. However, the Boltysh impact event is inferred to have occurred 2 to 5 ka before the K-Pg boundary because sediments overlying the Boltysh impactites suggest a short period of palynological recovery followed by a “barren zone” that was hypothesized to correspond to the end-Cretacous extinction (*17*). Therefore, refining temporal relationships between the Boltysh impact, the K-Pg boundary, eruption of the Deccan Traps, and the Chicxulub impact event is crucial for a comprehensive understanding of this pivotal point in Earth history.

Here, we present a precise age for Boltysh, which has been obtained by ^40^Ar/^39^Ar analyses of impact melt rocks from the structure. The impact melt rocks were coirradiated and analyzed with sanidine from the IrZ coal [66.04 ± 0.02/0.08 Ma, 2σ, analytical/systematic uncertainty, ^40^Ar/^39^Ar (*3*, *4*)], which is the stratigraphically closest dateable material to the K-Pg boundary, located in Montana, USA.The IrZ sanidine was used as the fluence monitor (age standard) to provide maximum temporal resolution of the sequence of events. By analyzing these materials under the same experimental conditions, we have minimized sources of uncertainty in the age comparison [e.g., (*18*)].

### Geological setting of the Boltysh impact structure

The Boltysh impact structure, Ukraine (48°45′N; 32°10′E), is approximately 24 km in diameter with a 6-km-diameter central uplift (*19*). Boltysh is located in the central part of the Ukrainian Shield, where the target rocks are Precambrian granites and granitic gneisses [1550 to 2220 Ma (*19*)]. The structure is now buried beneath >500 m of post-impact sediments (*19*, *20*).

The age of the Boltysh impact structure has been previously constrained using stratigraphy, palynology, fission track, K-Ar, and ^40^Ar/^39^Ar geochronology, with results ranging from ~56 to 173 Ma (table S1) (*14*, *17*, *21*–*27*). The most precise ages are derived from the ^40^Ar/^39^Ar method and palynology.

Previous single-grain step-heating and in situ ultraviolet laser spot ^40^Ar/^39^Ar ages from impact glasses and microcrystalline melt rocks yielded an impact age of 65.80 ± 0.64/0.68 Ma [2σ, systematic uncertainty (*14*)]. This age places Boltysh within uncertainty of the Chicxulub impact structure (66.04 ± 0.05/0.10 Ma) and the K-Pg boundary (66.04 ± 0.02/0.08 Ma) as determined by ^40^Ar/^39^Ar step-heating analysis of impact-generated glass spherules and IrZ sanidine, respectively (*3*, *4*).

A study discussing the age of Boltysh on the basis of palynology and carbon isotopes (δ^13^C) recorded in crater fill deposits (*17*) suggested that flora that had colonized the crater had subsequently been devastated by the K-Pg extinction. Similar early mid-successional communities can develop within 2 to 5 ka between lava flows in large igneous provinces (*28*) and in modern lava fields (*29*). On the basis of comparison with such interlava flow communities, Jolley *et al*. (*17*) suggested a likely interval of 2 to 5 ka between the Boltysh impact and devastation that produced the barren zone. The conclusion therefore was that the Boltysh event predated the Chicxulub impact, and the K-Pg boundary, by ~2 to 5 ka (*17*). Two relatively large impact events this close together in time (within ~5 ka) is improbable according to models of the impactor flux rate on Earth (*30*), which predict that 4 ± 2 craters ≥ 20 km in diameter form on land every 5 Ma (*30*), corresponding to one Boltysh-sized impact every ~0.83 to 2.5 Ma.

**Fig. 1:**
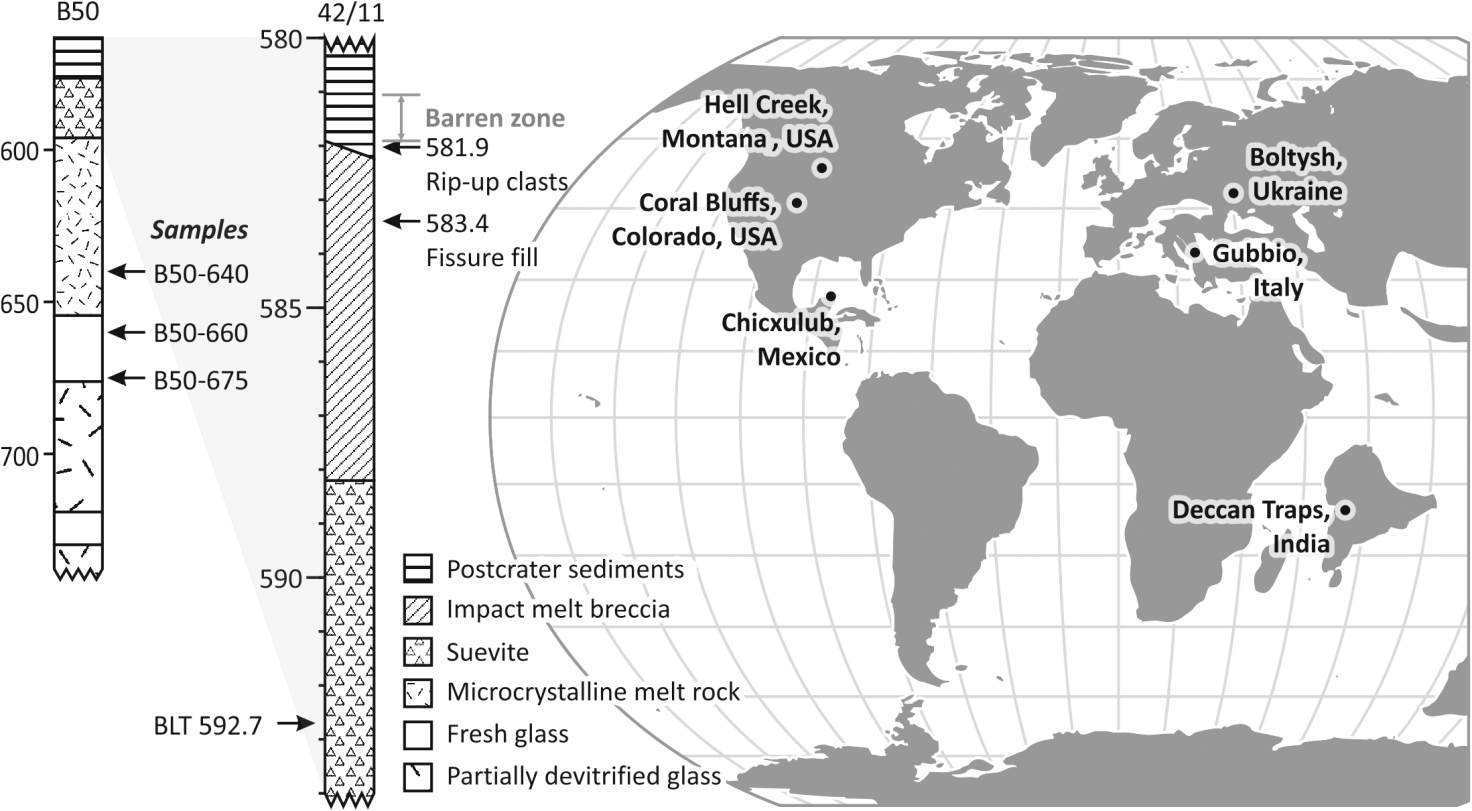
Lithology of two Boltysh drill cores (42/11 and B50) and approximate locations of sites related to the K-Pg boundary at 66 Ma. (**A**) Lithologic columns for two cores recovered from the Boltysh impact structure. Modified from (*31*) and (*19*). (**B**) Palaeomap of locations relevant to events around the K-Pg boundary. Palaeomap generated by the gplates Paleomap Maker using the model of Muller2016 with a Robinson projection (*70*).

Samples used in this study come from two drill cores: cores B50 and 42/11 (Fig. 1). Core B50 was drilled ~3.5 km from the center of the structure (*19*). This core recovered postimpact sediment, impact melt-bearing breccia, microcrystalline melt rock, and glassy melt rock [Fig. 1 (*19*)]. Core 42/11 was drilled to the west of the central peak in the deepest part of the crater [48°56′18″, 32°12′24″ (*17*)]. It recovered 596 m of core; the lowest ~16 m are impactites [impact melt-bearing breccia, sometimes called suevite or suevitic breccia (*31*)], and the remainder is postimpact sediment [Fig. 1 (*17*)]. Most studies conducted on core 42/11 thus far have focused on the postimpact sediment infilling the crater. Gilmour *et al.* (*32*) identified cyclicity in the δ^13^C values recorded in the crater fill sediments, which they associated with ~30-m-thick precession cycles.

### Geological setting of the IrZ sanidine

IrZ sanidine is so named because it comes from the iridium-rich (Ir) layer of the Z-coal, which is the basal coal of the Paleogene in the Western Williston Basin, northeast Montana, USA. The sanidine crystals are in a tephra preserved in a coal layer at the contact between the upper Hell Creek Formation (Cretaceous) and the Tullock member of the lower Fort Union Formation (Paleogene). The IrZ sanidines and sanidines in other coal layers have formed the basis of chronostratigraphy for the area, providing bracketing ages for the surrounding beds and fossils [e.g., (*33*, *34*)]. The K-Pg boundary is locally represented in this area by a clay horizon, which contains an iridium anomaly, shocked quartz, and spherules (*33*) that confirm its association with a hypervelocity impact event.

The IrZ tephra is the closest stratigraphic proxy for the K-Pg boundary. The coal in which it occurs is ≲ 1 cm above the impact-generated clay layer (*33*, *35*), making it the stratigraphically closest datable material to the impact signals (e.g., Ir anomaly) and the bio-stratigraphic K-Pg boundary (*4*). Therefore, the currently accepted age for the K-Pg boundary [66.04 ± 0.02/0.08 Ma, 2σ, analytical/systematic uncertainty, (*3*, *4*)] comes from ^40^Ar/^39^Ar geochronology of sanidine from the IrZ coal tephra (*4*, *33*, *36*) and agrees with the age of the Chicxulub impact as determined by ^40^Ar/^39^Ar geochronology of impact-generated glass spherules [66.04 ± 0.05/0.10 Ma, 2σ (*4*, *9*)].

## RESULTS

Four samples of Boltysh impact melt rocks were analyzed, three from core B50 (B50-640, B50-660, and B50-675), and one from core 42/11 (BLT-592.7). Sample names correspond to depth within the core, and locations are indicated on Fig. 1.

### Petrography

#### Microcrystalline melt rock

**Fig. 2:**
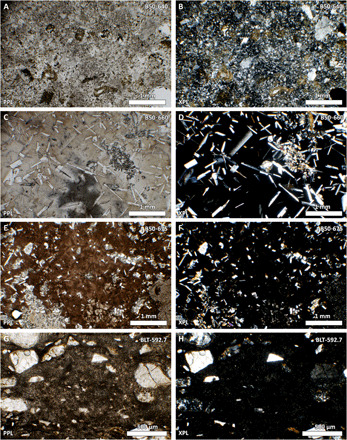
Transmitted light photomicrographs of impact melt rocks used in this work. (**A**) A representative portion of sample B50-640 (microcrystalline melt rock). (**B**) The same field of view as (A), between crossed polarizers. (**C**) A representative portion of sample B50-660 (glassy impact melt rock). (**D**) The same field of view as (C), between crossed polarizers. (**E**) A representative portion of sample B50-675 (glassy impact melt rock). (**F**) The same field of view as (E), between crossed polarizers. (**G**) A representative portion of sample BLT-592.7 (microcrystalline impact melt clast). (**H**) The same field of view as (G), between crossed polarizers. PPL, plane-polarized light; XPL, between crossed polarizers. Photo credit: Annemarie E. Pickersgill, University of Glasgow.

Sample B50-640 is fairly homogenous, composed of a groundmass of microcrystalline K-rich feldspar [Or_50–66_, according to Grieve *et al.* (*19*)], with ~20% feldspar phenocrysts, ~10% quartz clasts, and ~ 5% chlorite (Fig. 2). Both the feldspar phenocrysts and groundmass have features indicative of rapid cooling, such as swallow-tail and skeletal textures.

#### Glassy impact melt rocks

Sample B50-660 has a groundmass of light brown glass with local areas of semi-perlitic fracturing. Brown coloration is clearer in some areas of the glass than in others. In particular, the glass is clear in haloes around the pyroxene crystals (Fig. 2). Approximately 15% of the glass is a dark gray color when viewed at low magnification with transmitted light. Clear haloes in the glass are concentrated around these dark gray areas. Higher magnification shows that these dark gray areas are concentrations of very thin apparently tubular features within the glass. Concentrations of these features vary significantly within different areas of the glass. Plagioclase laths approximately 250 to 500 μm long and 50 to 100 μm wide comprise ~15% of the rock. Some show a skeletal texture and/or swallow tail terminations. Pyroxene makes up ~5% of the rock, largely as aggregates of 100-μm-size relatively equant crystals (Fig. 2).

Sample B50-675 has a dark brown groundmass that is locally lighter around quartz clasts and contains areas with semi-perlitic fracturing. In plane polarized transmitted light, the groundmass appears to be glass; however, when viewed between crossed polarizers, partial birefringence shows that the glass is at least partially devitrified (Fig. 2). Plagioclase laths ~100 to 500 μm long by 50 μm wide comprise ~10% of the rock. Some show a skeletal texture and/or swallow tail terminations. Pyroxene crystals make up ~5% of the rock; they commonly occur in aggregate groups of 100 μm–by–50 μm elongate crystals, which are frequently concentrated around the edges of quartz clasts. Glass is locally lightened in areas surrounding pyroxene crystals. Clasts make up 5 to 10% of the rock and are typically ballen silica or sieve-textured plagioclase.

#### Impact melt clast in breccia

Sample BLT-592.7 from core 42/11 comes from the Middle Unit (gray impact melt-bearing breccia or “suevite”) of (*31*). The sample is mainly one large dark impact melt clast extracted from the gray impact melt-bearing breccia (Fig. 2). The clast is black in the interior with a red rim ~1 mm wide. The impact melt contains ~25% lithic and mineral clasts up to 4 mm in size, most of which appear rounded and have red rims usually <0.5 mm in width. There are one or two very small vesicles. The impact melt is formed of small acicular, radiating crystals, with flow textures defined by variations in darkness when viewed in transmitted light (Fig. 2).

### ^40^Ar/^39^Ar analyses

Forty-five single grains of IrZ sanidine were analyzed by laser total fusion. The age of IrZ has previously been referenced against international standard Fish Canyon sanidine (FCs) (*4*). Using IrZ as the fluence monitor, the weighted mean age of these crystals was used to calculate a *J* value with the age set to 66.04 ± 0.02/0.08 Ma (2σ), the age of the IrZ sanidine determined by (*4*). This approach allowed for determination of a precise time difference (Δ*t*) between the Boltysh impact and the K-Pg boundary.

Groundmass separates of K-rich feldspar (B50-640 and BLT-592.7) and impact glass (B50-660 and B50-675) from the four impact melt rocks described above were analyzed by CO_2_ laser step-heating. Sample B50-640 (microcrystalline melt rock) yielded six plateau ages with a weighted mean age of 65.37 ± 0.17 Ma (2σ, analytical precision). Sample B50-660 (glassy impact melt rock) yielded two plateau ages with a weighted mean age of 92.45 ± 0.41 Ma (2σ, analytical precision). Sample B50-675 (glassy impact melt rock) yielded four discordant age spectra, generally starting with old low-temperature ages falling to younger high-temperature ages and sometimes rising again to slightly older ages in the final steps. These saddle-shaped age spectra are a characteristic indicator of the presence of extraneous ^40^Ar [e.g., (*37*–*40*)].

Sample BLT-592.7 (impact melt clast in breccia) yielded two plateau ages and two discordant age spectra. The weighted mean of the two plateau ages is 65.53 ± 0.31 Ma (2σ, analytical precision). All ages are summarized in table S3, individual age spectra and isotope correlation plots can be found in figs. S1 to S6, and complete datasets are presented in the data tables in the Supplementary Materials.

## DISCUSSION

### The age of the Boltysh impact structure

**Fig. 3:**
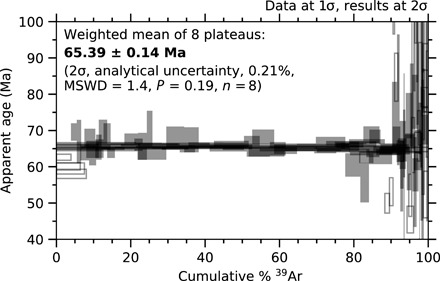
Age spectra plots of eight step-heating analyses from two impact melt rocks (B50-640 and BLT-592.7) that yielded plateau ages. Weighted mean of plateau ages is 65.39 ± 0.14/0.16 Ma (2σ, analytical/systematic uncertainty, 0.21/0.24%, MSWD = 1.4; *P* = 0.19; *n* = 8). Steps included in the plateau are filled; steps not included in the plateaus are outlines only. Individual age spectra and isotope correlation plots can be found in figs. S1 to S6.

**Fig. 4:**
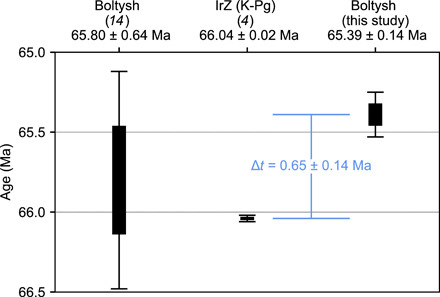
Age comparison chart of results of this study with the K-Pg boundary [defined by the age of the IrZ sanidine from (*4*)] and with the previous ^40^Ar/^39^Ar Boltysh age from (*14*). The revised age from this study overlaps with previous age constraints for Boltysh but is more precise. The higher precision demonstrates that the Boltysh impact occurred 0.65 ± 0.14 Ma (2σ) after the end-Cretaceous mass extinction. Boxes indicate 1σ; error bars indicate 2σ uncertainties.

The weighted mean of the eight plateau ages (Fig. 3) from two crystalline melt rocks (six from B50-640 and two from BLT-592.7) yields an age for the Boltysh impact structure of 65.39 ± 0.14/0.16 Ma [2σ, analytical/systematic uncertainty, 0.21%, mean square weighted deviates (MSWD) = 1.4, *P* = 0.19, *n* = 8; table S3]. Systematic uncertainty for age interpretations is reported to allow interchronometer comparisons; analytical uncertainties are used for determination of intrachronometer durations. This age overlaps with, but is more precise than, the previous ^40^Ar/^39^Ar age determined for Boltysh of 65.80 ± 0.64/0.68 Ma [2σ, analytical/systematic uncertainty, Fig. 4 (*14*)]. The improved precision shows that the Boltysh impact structure postdates the K-Pg boundary by 0.65 ± 0.14 Ma (2σ) (Fig. 4).

The improved precision of our age is a result of the combination of increased precision on individual isotope (ion beam) mass spectrometer measurements and greater number of plateaus [we obtained eight plateaus compared to four in Kelley and Gurov (*14*)]. The different precision was not due to different instrumentation or samples. We used the same mass spectrometer as Kelley and Gurov (*14*) and similar rock types. They analyzed two samples of microcrystalline melt rock and one of devitrified glass from core B50 (depths of 622, 652, and 710 m, respectively), and we used microcrystalline melt rocks from core B50 (640 m depth) and from core 42/11 (592.7 m depth).

Individual measurement uncertainties in the present study were reduced compared to those in Kelley and Gurov (*14*) because the achievable precision of ^40^Ar/^39^Ar geochronology has increased by an order of magnitude in the intervening 18 years. These improvements are due to advances in mass spectrometry (data treatment, number of calibrations of ^40^Ar/^36^Ar, number of background measurements, duration of measurements, electronic stability via uninterruptible power supplies/voltage smoothing, and greater number of fluence monitor measurements) and reduction in uncertainties associated with, for example, the atmospheric ^40^Ar/^36^Ar ratio (formerly ±1.5, now ±0.3). To minimize limitations inherent to mass spectrometry (e.g., mass discrimination and detector nonlinearity), it is desirable to use fluence monitors whose radiogenic ^40^Ar to ^40^K (^40^Ar*/^40^K) ratios are similar to those of the samples. We therefore further reduced uncertainty in individual age calculations by using IrZ sanidine as the fluence monitor as it both dates the K-Pg boundary (a key comparison for this study) and has a similar ^40^Ar*/^40^K ratio to the Boltysh impact melt rocks. It is worth noting that for the current study, the age determined by Kelley and Gurov (*14*) was recalculated using the refined decay constants and monitor ages of Renne *et al.* (*15*, *16*), so improved uncertainties in this study are not a result of improved uncertaintites in the decay constants or monitor ages, but rather in improved analytical and statistical uncertainties on the samples from Boltysh.

Our refined age disagrees with the conclusion by Jolley *et al.* (*17*) that the Boltysh impact predated the K-Pg boundary by 2 to 5 ka. The revised Boltysh age also means that reinterpretation of the crater-fill sedimentary sequences and palynology is necessary. The barren zone discussed in (*17*, *41*) is likely an effect of the Boltysh event itself and not a result of sterilization by Chicxulub as inferred by Jolley *et al*. (*17*). Unfortunately, much of core B50 has been lost, and a high-resolution palynology/isotopic study of the crater fill from that core does not exist (*42*). However, coupling the revised age to the palynology and sedimentology work done on core 42/11 [e.g., (*17*, *32*)] suggests the following sequence of events. Soon after the impact, patchy low-lying wet areas (i.e., marshes and similar) developed within the crater as evidenced by early mid-successional vegetation hosted by the lowest two palynoflora-containing samples. These two samples were recovered from fissure fill in the impactites (583.4 m) and rip-up clasts in overlying sediments (581.9 m) as described in (*17*). Subsequent flooding of the crater, as evidenced by turbidite deposits at 579.6 m, probably destroyed the primary colonizers on the crater floor and created the barren zone. During this time, material being deposited was sourced from the periphery of the crater and nearby ejecta, which had been mobilized by reorganization of local drainage. Subsequent colonization of crater fill was similar to K-Pg recovery patterns observed in the Midwest USA [e.g., (*17*, *32*)]. However, in the case of Boltysh, the floral recovery was from devastation caused by flooding, not a K-Pg–specific event. A side-by-side comparison of the previous interpretation (*17*) and the interpretation presented here can be found in table S4.

In addition to the refined age for Boltysh, the above interpretation is supported by the notable absence in Boltysh cores of the diagnostic boundary clay and geochemical anomalies associated with other K-Pg sites, although a postimpact crater lake, formed within 5 ka of impact (*17*), would presumably have been an ideal environment for deposition and preservation. This is particularly true given the proximity of Boltysh to Gubbio, Italy (Fig. 1), which preserves a detailed record of the K-Pg boundary and global iridium anomaly in a claystone layer (*43*). Unfortunately, we should not expect to find a record of the Boltysh impact at Gubbio, due to the smaller size of Boltysh compared to Chicxulub. Ejecta from Boltysh is estimated to have been only about 10 μm thick at the distance of Gubbio [using a transient crater diameter of ~16 km and a distance between the two locations of ~1600 km (*44*)].

### Extraneous ^40^Ar

Both glass samples yielded old ages, with two plateau ages and four discordant saddle-shaped age spectra. Saddle-shaped spectra are indicative of extraneous ^40^Ar either incorporated via fluids or inherited from a target rock that was incompletely degassed during impact. The older integrated and plateau ages derived from the glassy impact melt rocks are similar to ^40^Ar/^39^Ar results obtained from glassy impact melt rocks by Kelley and Gurov (*14*) and in agreement with the interpretation of Grieve *et al*. (*19*) that the impact melt would have been too viscous to allow for extensive species migration. The presence of extraneous ^40^Ar is supported by a supra-atmospheric initial trapped ^40^Ar/^36^Ar ratio wen data are plotted on isotope correlation plots (figs. S4 and S6). Another possible explanation for the older measured ages is ^39^Ar recoil loss during neutron bombardment in the nuclear reactor. However, all analyzed material was >250 μm in size, and the glass appears coherent petrographically, leaving minimal pathways for ^39^Ar loss.

It was hoped that impact glass from this mid-sized structure might prove more amenable to ^40^Ar/^39^Ar age determination than impact glass from smaller impact structures such as Gow Lake and Tswaing (*39*, *40*). Higher water content in a melt promotes argon diffusion (*45*); however, despite the presence of perlitic fracturing (a sign of hydrated melt), it seems that the glassy melt at Boltysh was too silicic, and therefore still too viscous, to allow for argon to be completely degassed during melting. On the other hand, the microcrystalline melt rock from sample B50-640 cooled over a longer period of time (as evidenced by the formation of crystals) than the glassy impact melt rocks (as evidenced by quench textures such as glass and quench crystallites). Thus, there was more time for ^40^Ar to diffuse out of the melt so that it was not incorporated into the crystals [e.g., (*37*)].

### Implications of a revised age

The Boltysh impact structure formed 65.39 ± 0.14/0.16 Ma ago (2σ), postdating the K-Pg boundary and the Chicxulub impact event by 0.65 ± 0.14 Ma (2σ). This refined age for Boltysh is consistent with the modeled terrestrial cratering rate for impacts with crater diameter ≥ 20 km on land (*30*). This cratering rate predicts one impact every ~0.83 to 2.5 Ma and suggests that more than 650 similar sized impacts have occurred on land during the Phanerozoic era.

This study raises questions about the effect that a midsize hypervelocity impact might have on a vulnerable global climate, if it formed before the climate had fully recovered from a large hypervelocity impact (Chicxulub), and while the climate was experiencing forcing from ongoing flood basalt volcanism in the Deccan traps (*10*, *13*, *46*). Here, we link the Boltysh sediment record to events occurring in the Deccan Traps and climate archives preserved in marine and terrestrial settings.

#### The Boltysh sediment record

Hyperthermal events are common throughout the Paleocene [e.g., Paleocene-Eocene Thermal Maximum, Lower C29n, and Dan-C2 (*47*)]. Hyperthermals recorded throughout the geological record are associated with increases in global temperature, changes in *p*CO_2_, ocean acidification, mass extinctions, and changes in hydrology [e.g., (*48*)]. The Boltysh crater lake sediments record a carbon isotope excursion previously linked to the Dan-C2 hyperthermal event (*17*, *49*, *50*), based on the previous age of the crater and the cyclic nature of the sediments.

The cyclic nature of sediments found within the Boltysh impact crater has been linked to Milankovitch control by Gilmour *et al*. (*51*). They conducted multitaper spectral analysis of linearly interpolated raw δ^13^C data for total organic carbon (*51*). That analysis highlighted the presence of cycles with wavelength of ~30 m. Cycle counting using both raw and filtered data show the presence of 8.5 cycles of ~30 m. The Boltysh record is analogous to high-resolution records obtained from Quaternary lake sediments with fine structure and variability reflecting lacustrine rather than marine cyclicity. The cyclostratigraphy from δ^13^C isotopes in the Boltysh core has been used to correlate events recorded in core 42/11 with events in other isotope archives (e.g., marine cores).

**Fig. 5:**
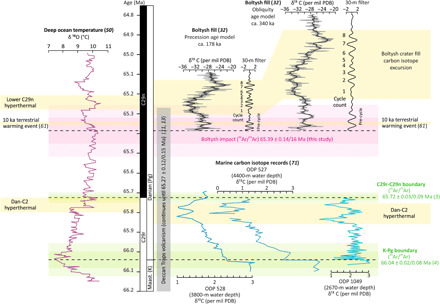
Time scale correlating events surrounding the K-Pg boundary/Chicxulub impact, the Boltysh impact, and the Dan-C2 and Lower C29n hyperthermals, calibrated using the age models of (*3*, *4*). From left to right: (i) The recent deep ocean temperature record from benthic foram δ^18^O isotope record of (*50*); (ii) the carbon isotope record from Boltysh crater fill (core 42/11) of (*32*) calibrated using a precession age model (left) and an obliquity age model (right) (PDB, Pee Dee Belemnite standard); (iii) marine δ^13^C records from the Atlantic Ocean recording the Dan-C2 hyperthermal [Ocean Drilling Program (ODP) 527, 528, and 1049] (*71*); and (iv) the Deccan Trap age model reported in (*11*, *13*), volcanism continued until 65.27 Ma (*13*). Yellow bars indicate periods of warming as recorded in the oxygen and carbon isotope records. Slight offset between the Dan-C2 event in the carbon and oxygen isotope records is likely due to small differences in the age models applied to the different cores. In the Boltysh crater fill record, using the precession age model aligns the Lower C29n hyperthermal recorded at Boltysh with the Lower C29n hyperthermal of Barnet *et al.* (*50*). However, the Boltysh core provides a higher-resolution record of the hyperthermal than the deep ocean record. Note that data plotted below the start of the numbered cycles are of poor quality because the sedimentation rate in that part of the core is unclear (*17*). The short 10 ka terrestrial warming event recorded at Corral Bluffs, Colorado (*61*), is presented; however, we consider this most likely temporally misaligned (see section: Relationship to Corral Bluffs for discussion).

Based on the age of the C29r/C29n boundary and the previous age for Boltysh, the isotope records recovered from Boltysh lake sediments were previously interpreted to record the Dan-C2 hyperthermal, which occurred immediately before the C29r/C29n boundary, as recorded by deep sea δ^18^O records (*17*, *49*, *50*). Previous estimates for the C29r/C29n boundary from various techniques range from 65.8 to 65.6 Ma, all of which predate the Boltysh impact event by several hundred thousand years. However, recent geochronology has recalibrated the post K-Pg geomagnetic time scale dating the C29r/C29n boundary to 65.72 ± 0.03/0.09 Ma (*3*). Coupling this age with the revised age for the Boltysh impact crater presented here (65.39 ± 0.14/0.16 Ma), it is clear that Boltysh postdates the Dan-C2 hyperthermal by ~0.4 Ma (Fig. 5). The Boltysh crater lake sediments thus record a later terrestrial carbon isotope excursion when plotted using the time scale of (*3*). The timing of this excursion depends on whether the cyclic nature of the Boltysh crater sediments (*41*) is recording a precession (21 ka) or obliquity (40 ka) periodicity (Fig. 5). If the precession age model is applied, then the carbon isotope excursion recorded by the Boltysh crater lake sediments shows temporal coincidence with the Lower C29n hyperthermal as recorded in the deep ocean benthic foram record (*50*). This conclusion indicates that the precycle sedimentation in the Boltysh core was deposited rapidly following the impact event [e.g., Fig. 5 (*32*)]. In addition, when using the precession age model, the duration of the excursions in the marine and terrestrial realms are similar (allowing for uncertainty in the age of the Boltysh impact structure). Whereas using the obliquity age model suggests that the hyperthermal lasted twice as long in the terrestrial realm than it did in marine settings, which is highly unlikely (*52*). We therefore favor the precession age model for the Boltysh crater lake sediments, which aligns the C29n hyperthermal isotope excursion in the marine and terrestrial realms.

The precession age model favored here agrees with the previous interpretation of Gilmour *et al*. (*51*) who observed ~8.5 cycles of 30 m thickness in the Boltysh sediment record. Thirty-meter-thick precession cycles are larger than those typically observed throughout the Quaternary, e.g., cycles of 10 to 15 m in African lake systems (*53*) and cycles of ~10 m in Eocene fluvial systems (*54*). However, thicker precession cycles have been reported in both Mesozoic and Paleozoic sediment archives. For example, a high-accommodation space Duckmantian-Bolsovian succession in the Netherlands and another from a medium-accommodation setting in the United States show individual precession-controlled cyclothems varying between 5 and 35 m thick (*55*). The thickness of these cycles may be due to interference of precession-, obliquity-, and eccentricity-driven sea-level fluctuations or, alternatively, to autocyclic or climate-controlled variations in sediment supply (*56*–*59*). The formation of the high-accommodation space Boltysh crater would have induced structural reorganization of local drainage patterns resulting in extremely high-sedimentation rates, enhanced by the surrounding fractured impact deposits that would have been easily eroded compared to consolidated lithologies. The high-sedimentation rate makes the site ideal for preservation of flora and fauna and for reconstruction of climate related events in the aftermath of the K-Pg mass extinction (*32*).

#### Relationship to the Deccan Traps

Although the eruption of the Deccan Traps is not directly related to the Boltysh impact event, Boltysh is coincident with the final stages of Deccan Trap volcanism (Fig. 5). The occurrence of a mid-sized impact event, during the recovery from the Chicxulub impact and during the waning of the Deccan Trap eruptions is unique in the geological record. The youngest (uppermost) lava flow associated with Deccan Trap volcanism (65.27 ± 0.12/0.15 Ma, 2σ analytical/systematic uncertainty) was erupted in C29n (Fig. 5) as part of the Mahabaleshwar Formation (*13*). This age is indistinguishable from the age of the Boltysh impact event and the age of the Lower C29n hyperthermal (Fig. 5).

#### Relationship to Corral Bluffs

Most detailed climate records of this time period come from marine records [e.g., (*49*, *60*)], but Boltysh is a terrestrial record, so it is of interest to compare to another terrestrial record, such as that recorded in the Corral Bluffs, Denver Basin, Colorado, USA (*61*). The revised age for the Boltysh impact event coincides with a tentative warming period of ~3°C over a duration of 10 ka at ~65.35 Ma as recorded in the Corral Bluffs (*61*). However, the chronostratigraphic framework applied to these sections is that of Fuentes *et al.* (*62*) who determined average sedimentation rates between each pair of dated tie-points using interpolated ages for three paleomagnetic chron boundaries [C30n/C29r, 66.398 Ma; C29r/C29n, 65.688 Ma; and C29n/C28r, 64.958 Ma; (*63*)], the K-Pg boundary ^40^Ar/^39^Ar age [66.04 Ma, (*4*)], and one chemical abrasion isotope dilution thermal ionization mass spectrometry ^206^Pb/^238^U date from Corral Bluffs (*61*). Recent work has recalibrated the C29r/C29n boundary to ~65.72 Ma, a change of ~90 ka (*3*) from (*63*). As such, there is potential that the warming event reported in (*61*) at 65.35 Ma is temporally misaligned if we consider the age model of Sprain *et al*. (*3*) to be accurate. The ~10 ka warming event likely correlates with the Lower C29n hyperthermal, which is apparently absent from the biotic recovery time scale in (*61*). However, because it is recorded by the Boltysh crater lake sediments and the deep ocean cores globally, there is no reason why the Lower C29n hyperthermal should not be recorded at Corral Bluffs.

#### Relationship to the Lower C29n hyperthermal

The carbon isotope excursion recorded in the Boltysh sediments has previously been linked to the Dan-C2 hyperthermal (*32*) due to temporal misalignment based on the previous interpretation of the sedimentology, but is now linked to the Lower C29n hyperthermal. At the 2σ uncertainty level, the age of the Boltysh impact event reported here (65.39 ± 0.14/0.16 Ma) is indistinguishable from the onset of the Lower C29n hyperthermal, recorded in the deep ocean δ^18^O records (Fig. 5). However, in the Boltysh core there is sediment between the impactites (crater formation) and the onset of the hyperthermal [as defined by δ^13^C records (*51*)]. The duration of this deposition is unknown as it occurs before the cyclic structure begins, and although there is some uncertainty in that part of the record, it is considered likely to be rapid infill following the impact event. We therefore consider the impact to have immediately preceded the Lower C29n hyperthermal, geologically speaking.

Compared to marine records of the Lower C29n hyperthermal, the Boltysh sediment core preserves a high-fidelity record of environmental change throughout the hyperthermal revealing a 3 per mil negative excursion in bulk organic matter (i.e., δ^13^C; Fig. 5). Changes in palynoflora throughout the Boltysh record are interpreted as a sequence of moisture availability cycles (*41*, *64*) that are aligned with the cyclicity observed in the carbon isotope record. The moisture availability cycles represent switching throughout the Lower C29n hyperthermal between winter wet (warmer and dryer) and mesic forest (cooler and wetter) vegetation biomes. Further interrogation of the Lower C29n hyperthermal recorded by the Boltysh sediment core may inform our understanding of vegetation and climate change at potentially human-relevant time scales, providing a direct analog for modern day climate change.

### Impact on climate

Hypervelocity impacts have been associated with both periods of global cooling and periods of global warming throughout geological time. Based on the complex effect of aerosols on global climate models, it is currently impossible to accurately determine whether the Boltysh impact contributed to destabilization of a climate that was recovering from the Chixculub impact event and ongoing Deccan Trap volcanism. Climate modeling by Fendley *et al*. (*13*) showed that the eruptive gas emissions of the lava flows at the end phase of the Deccan Traps were insufficient to directly cause multidegree warming; hence, a causal relationship with substantial climate warming during the post–K-Pg hyperthermal events would require an additional Earth system feedback. This study provides evidence of such an extra feedback in the form of the Boltysh impact event, whose age correlates with the age of the uppermost flow of the Deccan Traps (*13*) and the Lower C29n hyperthermal. Although it is unlikely that a relatively small impact would cause a hyperthermal event, the temporal coincidence of these events in the geological record is intriguing, and if there is a cause-effect relationship between Boltysh and the onset of the Lower C29n hyperthermal, then our data highlight the vulnerable nature of Earth’s climate to even small hypervelocity impacts during periods of recovery from catastrophic global events. Temporal coincidence alone is not enough to resolve cause and effect, but this study presents a hypothesis testable by future modeling studies.

## MATERIALS AND METHODS

### Core sampling

Sixteen samples were collected from Boltysh core 42/11 at the University of Aberdeen Core Store (table S2). Sample names correspond to depth within the core. Depth was measured from the edge of a box and/or from depth tick marks on core boxes. Samples used for ^40^Ar/^39^Ar analyses in this work are indicated on Fig. 1, and all samples are summarized in table S2.

Seven samples were provided from Boltysh core B50 and one from core B11475 by R. Grieve (table S2). R. Grieve obtained these samples during the 27th International Geological Congress field excursion 098 in 1984 (*19*). Samples used for ^40^Ar/^39^Ar analyses in this work are indicated on Fig. 1, and all samples are summarized in table S2.

All samples were examined using optical and scanning electron microscopy, and down-selected for ^40^Ar/^39^Ar analysis to one from core 42/11 (sample BLT-592.7) and three from core B50 (samples B50-640, B50-660, and B50-675).

### Optical microscopy

Transmitted light microscopy of polished thin sections from the core samples listed in table S2 was conducted on Zeiss Axioplan and Olympus BX41 petrographic microscopes. Image capture for the Zeiss Axioplan used a Nikon DS-Fi1 camera (2/3 inch, 5.24 megapixel CCD, 2560 × 1920 recording pixels) with Nikon NIS-Elements F3.0 software. Image capture for the Olympus BX41 used an Olympus DP25 camera (2/3 inch, 5.24 megapixel CCD, 2560 × 1920 recording pixels) with cell^B v2.8 software.

### Scanning electron microscopy

Backscatter electron imaging and qualitative electron dispersive x-ray spectroscopy (EDS) were conducted on thin sections from core 42/11. Thin sections from cores B50 and B11475 are covered sections and therefore unsuitable for scanning electron microscopy (SEM) analysis. Imaging and analysis used a field emission Zeiss Sigma SEM at the Imaging Spectroscopy and Analysis Centre (ISAAC), University of Glasgow. EDS used the Oxford Instruments AZTEC software and a silicon drift x-ray detector. Operating conditions for imaging and analysis of the carbon coated thin sections were high vacuum mode, probe current of 1 nA, accelerating voltage of 20 kV, and working distance of 8.5 mm.

### ^40^Ar/^39^Ar analyses

#### Step-heating sample preparation

Four samples (BLT-592.7, B50-640, B50-660, and B50-675) were prepared by crushing cut portions of individual samples (roughly 1 to 2 cm^3^) of impact melt rock in a disc mill. The crushed material was sieved and the 250- to 500-μm-size fractions were rinsed until clear in water in an ultrasonic bath and were then subjected to acid leaching, magnetic separation, and handpicking.

Impact melt rock from core 42/11 (sample BLT-592.7) was magnetically separated, and the magnetic fractions were leached ultrasonically for 20 min in 25% HNO_3_ and subsequently rinsed in deionized water and dried overnight in an oven (~70°C).

Impact melt rocks from core B50 (samples B50-640, B50-660, and B50-675) were leached ultrasonically for 5 min in 25% HNO_3_ and subsequently rinsed in deionized water and dried overnight in an oven (~70°C). They were then magnetically separated. The nonmagnetic fraction of sample B50-640 was again leached ultrasonically for 10 min in 25% HNO_3_, and subsequently rinsed in deionized water and dried overnight in an oven (~70°C).

The magnetic portions of samples B50-660, B50-675, and BLT-592.7 were handpicked under a binocular microscope, looking for unaltered grains with few or no inclusions. The handpicking of sample B50-640 was conducted on the nonmagnetic portion, as it appeared to have less alteration and contaminating material.

After picking, all samples were packaged into a 21-well aluminum irradiation disc, wrapped in aluminum foil, and sealed in a glass cylinder for irradiation. Crystals from the IrZ sanidine [66.04 ± 0.02/0.08 Ma, 2σ; (*4*)] were loaded symmetrically into the discs to act as a fluence monitor and provide a direct comparison between the Boltysh ages and the K-Pg boundary. IrZ sanidine was obtained from P. Renne (Berkeley Geochronology Centre). Samples were irradiated with fast neutrons for 50 hours at 1000 kW in the Cadmium-Lined in-Core Irradiation Tube (CLICIT) facility at the Oregon State University TRIGA Reactor. After irradiation, samples were left to cool for 8 months to allow short-lived radioisotopes produced during irradiation to decay.

#### Step-heating experiments

All ^40^Ar/^39^Ar analyses took place at the National Environment Research Council Argon Isotope Facility (NERC AIF), hosted by the Scottish Universities Environmental Research Centre (SUERC) in East Kilbride, Scotland. Aliquots of single grains were each loaded into individual wells in a 208-well steel laser pan. After loading the laser pan into the ultrahigh vacuum noble gas extraction line attached to a MAP 215-50 noble gas mass spectrometer, the pan was baked for 24 to 36 hours at 100°C to remove atmospheric contamination.

IrZ sanidine were analyzed by total fusion using a 55-W Teledyne Photon Machines Fusions 10.6 CO_2_ laser at 12.5% power for 20 s. IrZ sanidine was used for *J* value determinations. We are confident in using IrZ sanidine as a monitor mineral because it has been extensively studied in association with geochronological work on the K-Pg boundary (*4*, *33*) and has excellent reproducibility and precision. Forty-four of 45 analyses plotted in a coherent group, and the one analysis that did not plot in this group was discounted as an outlier based on a 3σ filter. The remaining 44 analyses were used to determine the *J* value for this irradiation (*J =* 0.0132897 ± 0.0000210, 2σ). This value reflects analyses of grains from three different wells in the same irradiation disc. When a *J* value is calculated for each well individually, the values all agree within 1σ, indicating that the horizontal variation in neutron flux over the diameter of the irradiation disc is negligible. Samples BLT-592.7, B50-640, B50-660, and B50-675 were analyzed by step-heating with a 55-W Teledyne Photon Machines Fusions 10.6 CO_2_ laser in step sizes detailed in the data tables in the Supplementary Materials.

Extracted gas fractions were subjected to 300 s of purification using two SAES GP50 getters (one at room temperature and one at 450°C) and a cold finger maintained at −95°C using a mixture of dry ice [CO_2(s)_] and acetone. Ion beams were measured using a MAP 215-50 noble gas mass spectrometer in peak-jumping mode with a measured sensitivity of 1.13 × 10^−13^ mol/V [e.g., (*65*)]. Isotope extraction, purification, extraction line operation, and mass spectrometry were fully automated. Backgrounds were measured after every two unknowns. Mass discrimination was monitored by analysis of air pipette shots after every five measurements.

#### Data analysis

For the purpose of these experiments, plateau ages were defined as containing a minimum of three contiguous steps overlapping at 2σ uncertainty, comprising >50% ^39^Ar released (*66*). Plateau ages were calculated using the mean weighted by inverse variance; plateau uncertainties were calculated using standard error of the mean (sem), but if MSWD > 1, then the sem was multiplied by the square root of the MSWD (*67*).

All data were regressed and handled using the Berkeley Geochronology Centre software MassSpec. ^40^Ar/^39^Ar ages were calculated using the decay constants from the optimization model of (*15*, *16*) and an IrZ sanidine monitor age of 66.04 ± 0.02/0.08 Ma (2σ) from (*4*). Mass discrimination values were determined using the atmospheric argon ratios of (*68*), which have been independently verified in (*69*).

## SUPPLEMENTARY MATERIALS

Supplementary material for this article is available at http://advances.sciencemag.org/cgi/content/full/7/25/eabe6530/DC1
